# Influence of biopolymers on the rheological properties of seafloor sediments and the runout behavior of submarine debris flows

**DOI:** 10.1038/s41598-021-81186-8

**Published:** 2021-01-15

**Authors:** Jun Kameda, Hamada Yohei

**Affiliations:** 1 Department of Earth and Planetary Sciences, Graduate School of Science, Hokkaido University, N10W8, Kita-ku, Sapporo, 060-0810 Japan; 2 Kochi Institute for Core Sample Research (KOCHII), Institute for Extra-cutting-edge Science and Technology Avant-garde Research (X-star), Agency for Marine-Earth Science and Technology, Nankoku, 783-8502 Japan

**Keywords:** Natural hazards, Solid Earth sciences

## Abstract

Submarine debris flows are mass movement processes on the seafloor, and are geohazards for seafloor infrastructure such as pipelines, communication cables, and submarine structures. Understanding the generation and run-out behavior of submarine debris flows is thus critical for assessing the risk of such geohazards. The rheological properties of seafloor sediments are governed by factors including sediment composition, grain size, water content, and physico-chemical conditions. In addition, extracellular polymeric substances (EPS) generated by microorganisms can affect rheological properties in natural systems. Here we show that a small quantity of EPS (~ 0.1 wt%) can potentially increase slope stability and decrease the mobility of submarine debris flows by increasing the internal cohesion of seafloor sediment. Our experiments demonstrated that the flow behavior of sediment suspensions mixed with an analogue material of EPS (xanthan gum) can be described by a Herschel–Bulkley model, with the rheological parameters being modified progressively, but not monotonously, with increasing EPS content. Numerical modeling of debris flows demonstrated that the run-out distance markedly decreases if even 0.1 wt% of EPS is added. The addition of EPS can also enhance the resistivity of sediment to fluidization triggered by cyclic loading, by means of formation of an EPS network that binds sediment particles. These findings suggest that the presence of EPS in natural environments reduces the likelihood of submarine geohazards.

## Introduction

Debris flows are a common mass movement process in both on-land and seafloor environments, but submarine flows characteristically exhibit longer run-out distances, often more than 100 km, and are thus an important sediment transport process on continental margins^1^. Because of their high velocity and long travel distances, submarine debris flows can cause severe damage to seafloor infrastructure such as communication equipment, power cables, and pipelines^2^. Understanding of submarine flow events is therefore a key issue for seafloor engineering. Submarine debris flows are also thought to trigger and/or enhance tsunamis. One notable example is the Storegga slide of offshore Norway, one of the largest documented seafloor mass-movement events^3–5^. During this slide, debris moved over a distance of more than 300 km and caused a catastrophic tsunami. A recent study demonstrated that the slide evolved more like a debris flow than a block slide^6^. The generation and post-failure run-out of debris flows are governed by the rheological properties of the sediment and seawater composition^7,8^, indicating that experimental determination of such properties is critical for precise assessment of geohazard risk in coastal areas.

Experiments have revealed that the rheological properties of seafloor sediments are dependent on several factors such as sediment composition, grain size, and physico-chemical conditions, such as pH, presence of electrolytes, and temperature^9–12^. Recent studies have also documented the importance of extracellular polymeric substances (EPS) generated by marine microorganisms for such rheological properties^13–15^. EPS are large biomolecules secreted by microbial cells, and attachment of the EPS matrix to sediment grains inhibits grain movement by forming a structured network (i.e., biofilm) that bonds the grains and affects sediment transport. Although those studies focused on the influence of EPS on surface sediment stability or bedform formation in shallow-marine environments, EPS are also thought to be supplied to the seafloor as marine snow^16^. More recently, Craig et al.^17^ reported on the effect of EPS on sediment gravity-flow dynamics. Based on analogue experiments, they demonstrated the inhibitory effect of EPS on the head velocity and the run-out distance of flow events, but the rheological properties of EPS-bearing sediments have not been well clarified. In particular, knowledge of such properties is a prerequisite for understanding of the post-failure behavior as well as the onset of such events. To investigate the rheological properties of EPS-containing sediments, we conducted rheological experiments on sediment suspensions mixed with xanthan gum, a system comparable to EPS-bearing seafloor sediments^17,18^. Based on the experimental results, we discuss the significance of EPS for the generation and post-failure run-out behavior of submarine debris flows.

## Flow behavior of sediments with and without EPS

**Figure 1:**
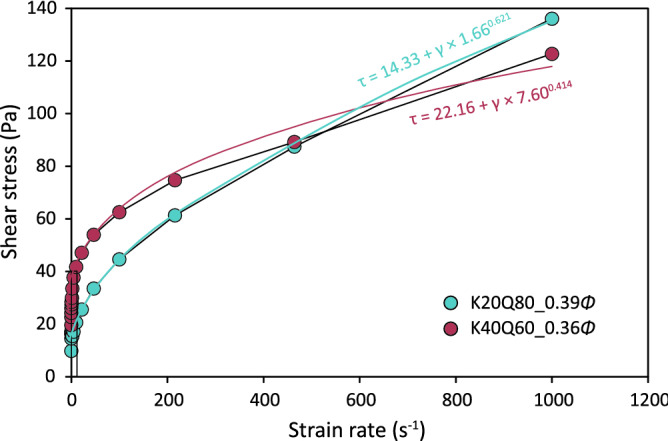
Typical flow curves for kaolinite–quartz suspensions without EPS.

The behavior of seafloor sediments was simulated by performing rheological experiments with a kaolinite–quartz mixture dispersed in NaCl solution (0.6 M) using a rheometer (HR-2 rheometer; TA Instruments). Details of the experimental procedures are provided in the Methods section. Initially, we examined the response of sediments without EPS under the applied strain rate $\dot{\gamma }$, which was increased stepwise from 0 to 1000 s^−1^ (flow ramp test). Typical flow curves (shear stress vs. strain rate) were thus obtained for suspensions without EPS for kaolinite 20% + quartz 80% at *Φ* = 0.39 (hereafter termed K20Q80_0.39*Φ*) and K40Q60_0.36*Φ*, where *Φ* denotes the solid volumetric fraction (Fig. 1).

The flow curves exhibit viscoplastic behavior and were reasonably fitted by a Herschel–Bulkley model (mostly with *R*^2^ > 0.99; Table S1):

1 $$\tau ={\tau }_{y}+\mu {\dot{\gamma }}^{n}$$

where $\tau$ is the shear stress, ${\tau }_{y}$ is the yield stress, $\mu$ is the dynamic viscosity, and *n* is the rate index. When *n* = 1, the equation represents Bingham rheology. Several previous studies have concluded that the nonlinear viscoplastic behavior of debris flows can be most appropriately described by the Herschel–Bulkley model^19,20^.

**Figure 2:**
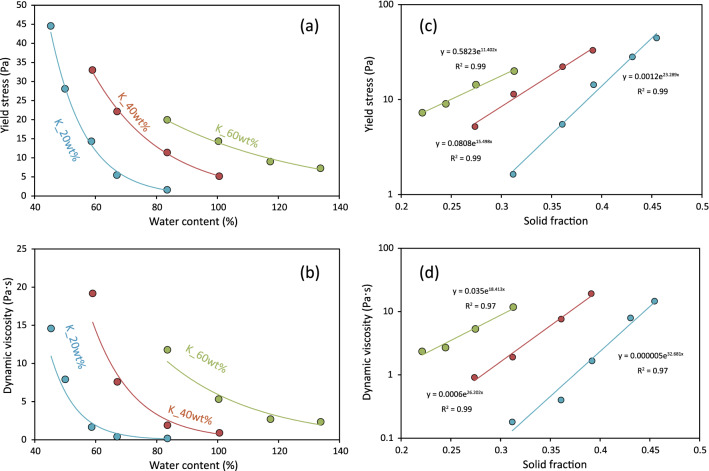
Plots of (**a**, **b**) rheological parameters versus water content, and (**c**) yield stress and (**d**) dynamic viscosity versus the solid fraction *Φ* of the suspension. Different kaolinite–quartz ratios are indicated by colored symbols.

The yield stress and dynamic viscosity of the suspensions are dependent on water content (Fig. 2a,b, also summarized in Table S1). Both the yield stress and the dynamic viscosity tend to decrease with increasing water content for a fixed kaolinite–quartz mixing ratio, whereas for the same water content, the two parameters increase with increasing kaolinite content in the solid component (Fig. 2a,b).

The yield stress and dynamic viscosity increase almost exponentially with increases in the solid fraction of the fixed solid composition (Fig. 2c,d), which is consistent with previous observations for a variety of landslide deposits^21,22^.

**Figure 3:**
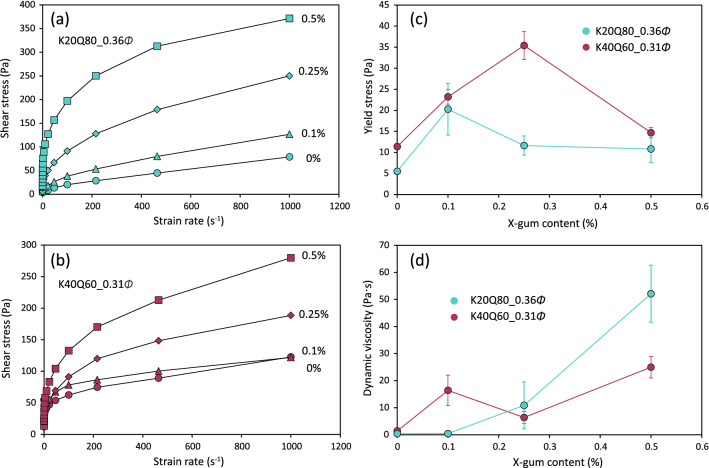
Flow curves for suspensions (**a**) K20Q80_0.36*Φ* and (**b**) K40Q60_0.31*Φ* with different xanthan gum contents, and plots of (**c**) yield stress and (**d**) dynamic viscosity versus xanthan gum content. Error bars denote the maximum and minimum values in three repeated tests.

Next, we examined the influence of xanthan gum on the rheological properties of the two suspensions (K20Q80_0.36*Φ* and K40Q60_0.31*Φ*). The flow curves also exhibit viscoplastic behavior that can be described by the Herschel–Bulkley model, but the shear stress successively increases with an increase in the xanthan gum content from 0.1% to 0.5% by weight (Fig. 3a,b). The rheological parameters obtained from these flow curves show somewhat complicated trends for different xanthan gum contents (Fig. 3c,d), but a dramatic change in the properties can be observed even when 0.1% of xanthan gum is added to the suspension. The yield stress more than doubles after addition of 0.1% xanthan gum, after which the stress decreases with increasing gum content, whereas the viscosity greatly increases by more than one order of magnitude from the original values after addition of 0.5% of xanthan gum. This result is not markedly different even after 24-h curing of the suspension (Fig. S1).

## Response to cyclic strain

**Figure 4:**
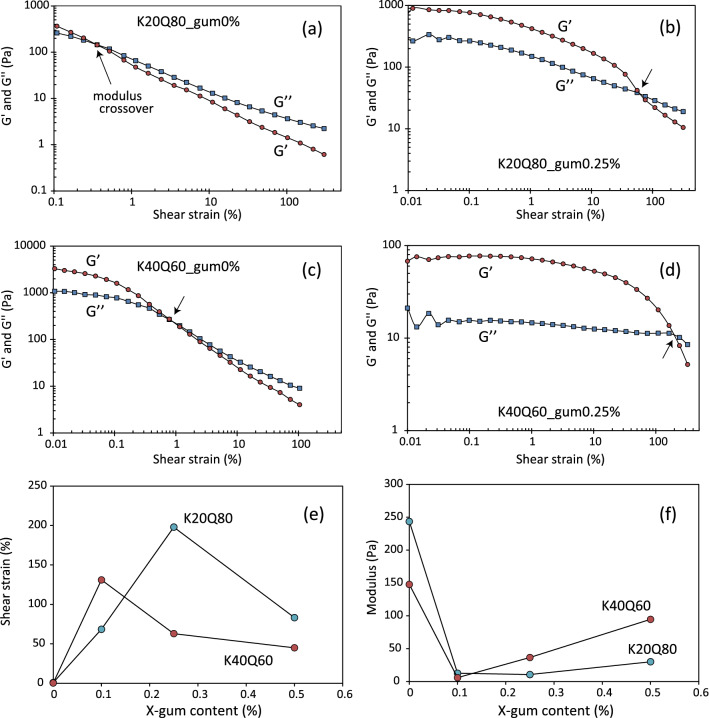
Results of amplitude sweep tests (**a**) K20Q80_0.36*Φ* _0% gum, (**b**) K20Q80_0.36*Φ*_0.25% gum, (**c**) K40Q60_0.31*Φ*_0% gum, and (**d**) K40Q60_0.31*Φ*_0.25% gum. *G*′ and *G″* are the storage and loss modulus, respectively. Also shown are crossover (**e**) shear strain and (**f**) modulus values as a function of xanthan gum content.

**Figure 5:**
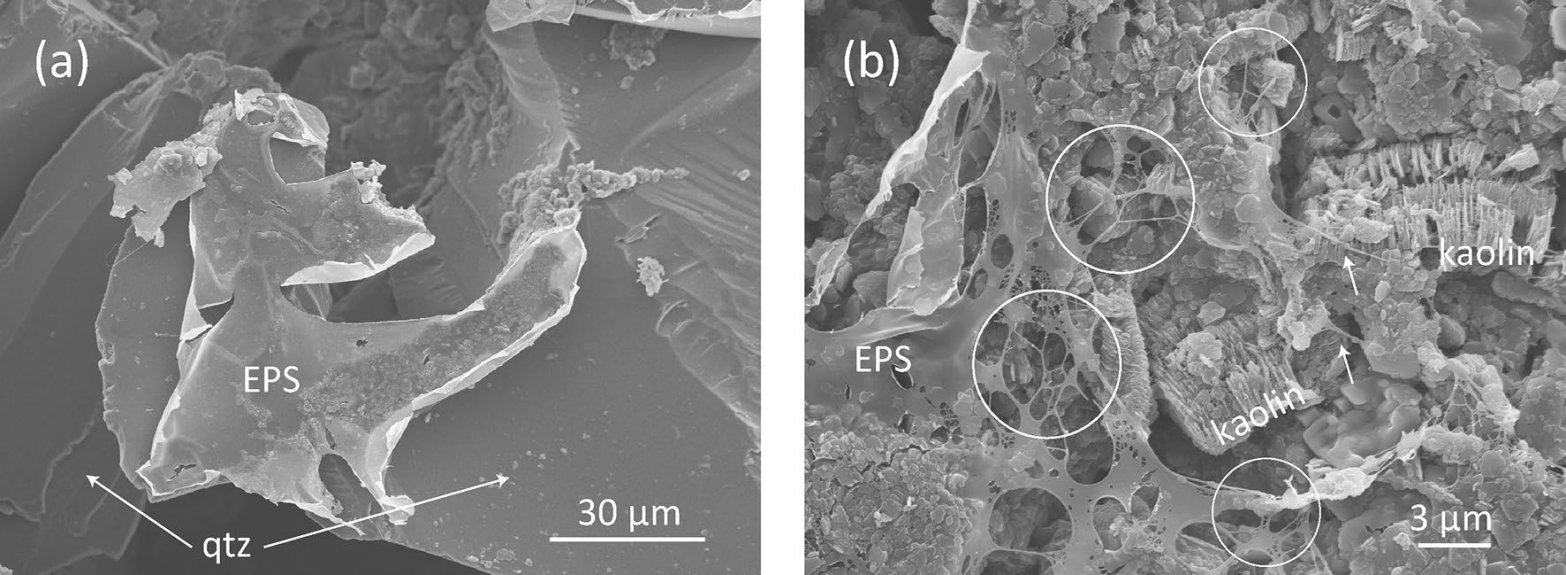
SEM images of dried samples (K40Q60_0.31*Φ*_0.5% gum). (**a**) Xanthan gum film physically bonded to quartz grains. Circles in (**b**) indicate typical fibrous textures of xanthan gum. Abbreviation: qtz = quartz.

Cyclic loading due to earthquake-induced coseismic ground shaking, wave action, storms and tides has been thought as a possible trigger for slope failure^1,2,23^. To examine the influence of EPS on the response to cyclic shear strain, oscillatory (strain sweep) testing was conducted on the K20Q80_0.36*Φ* and K40Q60_0.31*Φ* samples with and without xanthan gum (Fig. 4a–d). *G′* is the storage modulus, representing the elastic (solid) component of the response, and *G″* is the loss modulus, representing the viscous (liquid) component of the response. The relative sizes of *G′* and *G″* reverse at a certain strain, which is termed the modulus crossover. At smaller strain, *G′* > *G″*, indicating that the response is like that of a solid, whereas the reverse is true at higher strain, in which the response is liquid-like. When the sediments without xanthan gum are deformed, modulus crossover quickly appears at strain values of less than 1% (Fig. 4a,c), implying that the samples are susceptible to fluidization caused by oscillatory shear strain. However, the addition of xanthan gum dramatically increases the crossover strain, implying that the gum acts to stabilize the solid-like state. The crossover strain is highest at a gum content of 0.1%, and decreases at higher contents (Fig. 4e). Conversely, the modulus value markedly decreases after addition of 0.1% xanthan gum, and is then relatively stable or slightly higher at higher gum contents (Fig. 4f). These observations suggest that the solid-like state is achieved because the xanthan gum forms polymeric strands and gel surface coatings that physically bind solid particles to each other (i.e., biological cohesion)^15,24^. Scanning electron microscope (SEM) observation revealed that dried gum-containing samples exhibit fibrous to film-like xanthan gum attached to kaolin/quartz grains (Fig. 5). Of note, not only does the gum appear to increase the strength of the sediment up to a particular concentration, but its effects are mediated by the amount of clay in the suspension. These features might reflect differences in the polymerization products on the surfaces of quartz and clay grains (i.e., film or fiber) and hence their mechanical properties. In contrast, in the absence of xanthan gum the solid-like state may be supported by interparticle electrostatic bonding (i.e., physical cohesion)^15^ or direct contact of the solid particles, represented by higher modulus values, but the electrostatic bonding is easily broken and the sediment becomes fluidized by the application of small vibrations.

## Generation and post-failure run-out behavior of submarine debris flows

**Figure 6:**
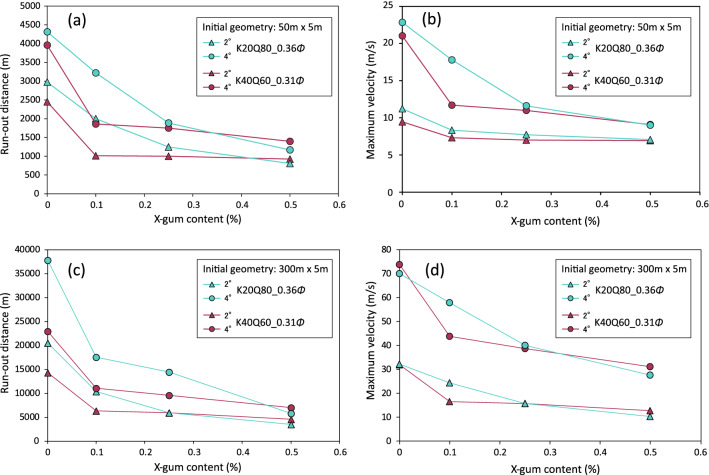
Run-out distances and maximum frontal velocities of two suspensions (K20Q80_0.36*Φ* and K40Q60_0.31*Φ*) on 2° and 4° slopes calculated using the BING program as a function of xanthan gum content. Initial geometries of the deposits are 50 m length × 5 m height (with a parabolic shape) in (**a**, **b**), and 300 m length × 5 m height in (**c**, **d**).

As demonstrated above, the rheological properties of sediments are dramatically influenced by adding even a small quantity (0.1%) of xanthan gum. In natural environments, the resistivity to cyclic strain, as observed in the oscillation tests, can enhance the resistivity of the seafloor slope to fluidization during coseismic shaking; thus, such behavior can reduce the possibility of liquefaction failure of the seafloor^1,2^. Even if the slope fails, strengthening of the sediment suspension will reduce the spreading distance of the debris. In this study, we carried out one-dimensional run-out modeling of debris flows based on the results from the flow ramp tests to examine the likely effects of EPS on debris flow behavior. Run-out distances and maximum frontal velocities of debris for the K20Q80_0.36*Φ* and K40Q60_0.31*Φ* suspensions are plotted in Fig. 6. Irrespective of the initial size of the deposits, both distance and velocity successively decrease with increasing content of xanthan gum, which is generally consistent with the results of analogue experiments conducted by Craig et al.^17^. At higher clay content (K40Q60), the distance falls markedly, to less than half of that without EPS, if 0.1% EPS is present (Figs. 6a,c), then slightly decreases with rising EPS content. At lower clay content (K20Q80), the effect of EPS is relatively moderate, but the distance eventually decreases by one-third if 0.5% EPS is present. The results for maximum frontal velocity of the debris are very similar to those for the run-out distance (Figs. 6b,d), implying that the impact force of debris on off-shore installations would also be reduced^25^. The general decrease in flow mobility with increasing EPS content is likely a result of the progressive strengthening of the suspension, as demonstrated in the flow curves (Figs. 3a,b). However, as described above, the individual rheological parameters exhibit rather complicated trends at EPS contents higher than 0.1%, and thus the reason for decreasing flow mobility may differ in each case.

It has been argued that factors such as geologic environment, sediment type, and flow mechanics affect mass-transport processes on the seafloor^1,2^. The rheological properties of seafloor sediments are governed by factors including clay content, grain size, water content, and physico-chemical conditions^9–12^, which is confirmed by the present experiment without EPS (Fig. 2). Our results indicate that EPS in seafloor sediments is another fundamental factor in the generation and post-failure behavior of submarine debris-flow events. The effect is clearly expressed even when a small quantity (~ 0.1%) of EPS is present. In shallow-marine environments, ~ 0.1% of EPS has been found to be pervasively distributed in marine sediments. Recent work by Craig et al.^17^ found that comparable amounts of EPS (average 0.139%, maximum 0.268%) are present in deep-sea sediments; thus, knowledge of where and how much EPS is distributed on the seafloor will be critical for precise understanding of submarine mass-transport processes. Finally, we note that biological polymers are now attracting growing interest as an environmentally friendly agent for on-land soil improvement^26,27^. The present findings will be crucial for future work exploring the applicability of geotechnical usage of biopolymers such as xanthan gum in the seafloor environment for risk management of submarine geohazards.

## Methods

### Material and sample preparation

We prepared kaolinite–quartz composite powders by mixing a kaolinite reference sample (KGa-1b; Clay Minerals Society) and crystalline quartz (silicon dioxide 199-00625; Wako Pure Chemical Industries Ltd.) at kaolinite weight fractions of 20, 40, and 60 wt%. The quartz powder is composed of angular crystals with a mean grain size of ~ 100 μm^28^. The reference kaolinite sample is composed of 97% kaolin (mostly kaolinite and trace dickite) with an average grain size of 20 μm and 3% anatase as an impurity^29^. The composite kaolinite–quartz powders were dispersed in NaCl solution (0.6 M) using a 50-ml polypropylene conical tube at different fluid contents (%; fluid weight/solid weight). The solid volume fraction *Φ* of each suspension was calculated from the grain densities of kaolinite and quartz (2.62 g/cm^3^ and 2.65 g/cm^3^)^30^. As a proxy for EPS in natural sediments, we added xanthan gum (11138-66-2; MP Biomedicals, LLC) to the suspensions (solid weight fractions 0.1%, 0.25%, and 0.5%), and blended these using a laboratory mixer (Vortex-Genie 2; Scientific Industries, Inc). The xanthan-gum-containing suspensions were cured for at least 2 h before the rheological tests.

## Rheological tests

Rheological tests on the suspensions were conducted using a HR-2 rheometer (TA Instruments) with a parallel-plate geometry. The rheometer contains an aluminum rotational upper plate with a diameter of 40 mm and a stationary lower Peltier plate kept at 7 °C. To avoid the influence of wall slip (i.e., slip between the suspension and the adjacent metal plate, rather than within the suspension), which is often a problem for rheometric experiments, waterproof sandpaper (φ = 125 μm) was attached to the upper plate. Samples were loaded into the 1.0-mm gap between the upper and lower plates. In this study, we performed two types of rheological tests: flow ramp tests and strain sweep tests. Prior to the flow ramp tests, samples were pre-sheared at a strain rate $\dot{\gamma }$ of 50 s^–1^ for 20 s to release the initial anomalous stress^31^. The rheological properties were then tested by measuring the shear stress $\tau$ under the applied $\dot{\gamma }$, which was increased in a stepwise manner from 0.01 to 1000 s^–1^. At each step, the averaged torque was acquired over 5 s after 15 s of shearing at the given strain rate following Coussot et al.^32^.

Strain sweep tests were conducted by applying an oscillatory shear strain with increasing amplitude, from 0.01% to 100%, at a constant frequency of 1 Hz (this frequency is commonly employed when measuring the resistance of soils to liquefaction in cyclic triaxial tests^33^). From the measurements, the frequency-dependent storage (*G′*) and loss-modulus (*G″*) components of the complex shear modulus G* = *G′* + i*G″* were determined^34^. All strain sweep tests were performed immediately after sample loading with no pre-shearing.

## Numerical Modeling of debris flows

For run-out modeling of debris flows, we carried out simulations of one-dimensional downslope flow using the BING program developed by Imran et al.^8^. We adopted the rheological parameters obtained from the above experiments as input parameters. We assumed that seafloor deposits of 50 m and 300 m length × 5 m height (with a parabolic shape) flowed down a slope with a dip of 2° or 4°. The cut-off time was set at 10 min for modeling of the 50 m × 5 m deposit, during which the frontal velocity of the debris flow slowed to typically less than one-tenth of the initial velocity. For the 300 m × 5 m deposit, the calculation was stopped when the frontal velocity of the debris flow decreased to < 0.5 m/s.

## Supplementary Information

Supplementary Information.

## References

[1] Hampton MA, Lee HJ, Locat J (1996). Submarine landslides. Rev. Geophys..

[2] Locat J, Lee H (2002). Submarine landslides: advances and challenges. Can. Geotech. J..

[3] Smith D (2004). The holocene storegga slide tsunami in the United Kingdom. Quat. Sci. Rev..

[4] Bondevik S, Svendsen JI, Mangerud J (1997). Tsunami sedimentary facies deposited by the Storegga tsunami in shallow marine basins and coastal lakes, Western Norway. Sedimentology.

[5] Bondevik S, Lovholt F, Harbitz C, Mangerud J, Dawson A, Svendsen J (2005). The Storegga slide tsunami comparing field observations with numerical simulations. Mar. Pet. Geol..

[6] Løvholt F, Bondevik S, Laberg JS, Kim J, Boylan N (2017). Some giant submarine landslides do not produce large tsunamis. Geophys. Res. Lett..

[7] Jiang L, LeBlond PH (1993). Numerical modeling of an underwater Bingham plastic mudslide and the waves which it generates. J. Geophys. Res..

[8] Imran J, Harff P, Parker G (2001). A numerical model of submarine debris-flow with graphical user interface. Comp. Geosci..

[9] Torrance JK (1999). Physical, chemical and mineralogical influences on the rheology of remoulded low-activity sensitive marine clay. App. Clay Sci..

[10] Jeong SW, Locat J, Leroueil S, Malet J-P (2010). Rheological properties of fine-grained sediment: the roles of texture and mineralogy. Can. Geotech. J..

[11] Kameda J, Morisaki T (2017). Sensitivity of clay suspension rheological properties to pH, temperature, salinity and smectite-quartz ratio. Geophys. Res. Lett..

[12] Kameda J, Hirauchi K (2018). Rheological properties of composite serpentine-brucite suspensions: Implications for mudflow behavior on forearc seamounts. Mar. Geol..

[13] Lundkvista M, Grue M, Friend PL, Flindt MR (2007). The relative contributions of physical and microbiological factors to cohesive sediment stability. Continent Shelf Res..

[14] Malarkey J (2015). The pervasive role of biological cohesion in bedform development. Nat. Commun..

[15] Parsons DR (2016). The role of biophysical cohesion on subaqueous bed form size. Geophys. Res. Lett..

[16] Decho AW, Gutierrez T (2017). Microbial extracellular polymeric substances (EPSs) in ocean systems. Front. Microbiol..

[17] Craig MJ (2020). Biomediation of submarine sediment gravity flow dynamics. Geology.

[18] Tan X, Hu L, Reed AH, Furukawa Y, Zhang G (2014). Flocculation and particle size analysis of expansive clay sediments affected by biological, chemical, and hydrodynamic factors. Ocean Dyn..

[19] Coussot P, Piau JM (1994). On the behavior of fine mud suspensions. Rheol. Acta.

[20] Huang X, Garcia M (1998). A Herschel–Bulkley model for mud flow down a slope. J. Fluid Mech..

[21] Malet JP, Rematre A, Maquaire O, Ancey C, Locat J, Rickenmann D, Chen CL (2003). Flow susceptibility of heterogeneous marly formations. Implications for torrent hazard control in the Barcelonnette basin (Alpes-de-Haute-Provence, France). Proceedings of the 3rd International Conference on Debris-Flow Hazards Mitigation.

[22] Malet J-P, Maquaire O, Locat J, Remaitre A (2004). Assessing debris flow hazards associated with slow moving landslides: methodology and numerical analyses. Landslides.

[23] Stigall J, Dugan B (2010). Overpressure and earthquake initiated slope failure in the Ursa region, northern Gulf of Mexico. J. Geophys. Res..

[24] Underwood GJC, Paterson DM (2003). The importance of extracellular carbohydrate production by marine epipelic diatoms. Adv. Bot. Res..

[25] Bruschi R, Bughi S, Spinazzè M, Torselletti E, Vitali L (2006). Impact of debris flows and turbidity currents on seafloor structures. Nor. J. Geol..

[26] Ayeldeen M, Negm A, El-Sawwaf M, Kitazume M (2017). Enhancing mechanical behaviors of collapsible soil using two biopolymers. J. Rock Mech. Geotech. Eng..

[27] Lee S, Chang I, Chung MK, Y. & Kee J. (2017). Geotechnical shear behavior of Xanthan Gum biopolymer treated sand from direct shear testing. Geomech. Eng..

[28] Oohashi K, Hirose T, Takahashi M, Tanikawa M (2015). Dynamic weakening of smectite-bearing faults at intermediate velocities: implications for subduction zone earthquakes. J. Geophys. Res. Solid Earth.

[29] Chipera SJ, Bish DL (2001). Baseline studies of the clay minerals society source clays: powder x-ray diffraction analyses. Clays Clay Miner..

[30] Deer WA, Howie RA, Zussman J (1992). An Introduction to the Rock-Forming Minerals.

[31] Heymann L, Peukert S, Aksel N (2002). Investigation of the solid-liquid transition of highly concentrated suspensions in oscillatory amplitude sweeps. J. Rheol..

[32] Coussot P, Leonov AI, Piau JM (1993). Rheology of concentrated dispersed systems in a low molecular weight matrix. J. Non-Newtonian Fluid Mech..

[33] ASTM Standard D5311. *Standard Test Method for Load Controlled Cyclic Triaxial Strength of Soil*. ASTM International, West Conshohocken, PA (2011).

[34] Barnes HA, Hutton JF, Walters K (1989). An Introduction to Rheology.

