# Supplementary material for: Influence of biopolymers on the rheological properties of seafloor sediments and the runout behavior of submarine debris flows
Source: Sci Rep. 2021 Jan 15;11:1493. doi: 10.1038/s41598-021-81186-8 (PMC7810891; doi:10.1038/s41598-021-81186-8)
Supplement: Supplementary file 1 — Supplementary Information. [file 41598_2021_81186_MOESM1_ESM.pdf]

Supplementary Materials for

Influence of biopolymers on the rheological properties of seafloor sediments and the runout behavior of submarine debris flows

Author #1: Jun Kameda, Department of Earth and Planetary Sciences, Faculty of Science, Hokkaido University, N10W8, Kita-ku, Sapporo, 060-0810, Japan, kameda@sci.hokudai.ac.jp

Author #2: Yohei, Hamada, Kochi Institute for Core Sample Research (KOCHII), Institute for Extra-cutting-edge Science and Technology Avant-garde Research (X-star), Japan Agency for Marine-Earth Science and Technology, Nankoku 783-8502, Japan, yhamada@jamstec.go.jp

This file includes:

Figs. S1 and Table S1.

Table S1 Compositions of the samples tested, experimental conditions, and rheological parameters determined from the experiments.

Experiment	Water content (%)	Solid fraction	Gum content (%)	Curing time (min)	HB yield stress (Pa)	HB viscosity (Pa·s)	Rate index	R^2
K20Q80	45.3	0.455	0	120	44.57	14.59	0.483	0.998
K20Q80	50.0	0.431	0	120	28.10	7.92	0.483	0.998
K20Q80	58.7	0.392	0	120	14.33	1.66	0.621	0.997
K20Q80	67.0	0.361	0	120	5.49	0.40	0.753	0.996
K20Q80	83.5	0.312	0	120	1.63	0.18	0.765	0.996
K40Q60	59.0	0.391	0	120	33.02	19.18	0.355	0.995
K40Q60	67.1	0.361	0	120	22.16	7.60	0.414	0.993
K40Q60	83.5	0.312	0	120	11.39	1.91	0.464	0.995
K40Q60	100.6	0.274	0	120	5.20	0.91	0.513	0.997
K60Q40	83.5	0.313	0	120	19.96	11.79	0.305	0.996
K60Q40	100.3	0.275	0	120	14.37	5.33	0.342	0.994
K60Q40	117.3	0.245	0	120	9.03	2.70	0.384	0.995
K60Q40	133.8	0.221	0	120	7.28	2.35	0.374	0.996
*K40_0.1%G	83.3	0.313	0.1	120	23.20	16.42	0.266	0.981
*K40_0.25%G	83.3	0.313	0.25	120	35.41	6.37	0.458	0.946
*K40_0.5%G	83.3	0.313	0.5	120	14.69	24.97	0.341	0.999
*K20_0.1%G	66.7	0.363	0.1	120	20.26	0.44	0.791	0.954
*K20_0.25%G	66.7	0.363	0.25	120	11.63	10.87	0.454	0.999
*K20_0.5%G	66.7	0.363	0.5	120	10.82	52.12	0.285	0.998
*KA40_0.5%G	83.3	0.313	0.5	70	10.28	12.66	0.423	0.999
*KA40_0.5%G	83.3	0.313	0.5	120	11.63	10.87	0.454	0.999
*KA40_0.5%G	83.3	0.313	0.5	1560	8.77	11.05	0.444	0.999

*average values in three repeated tests

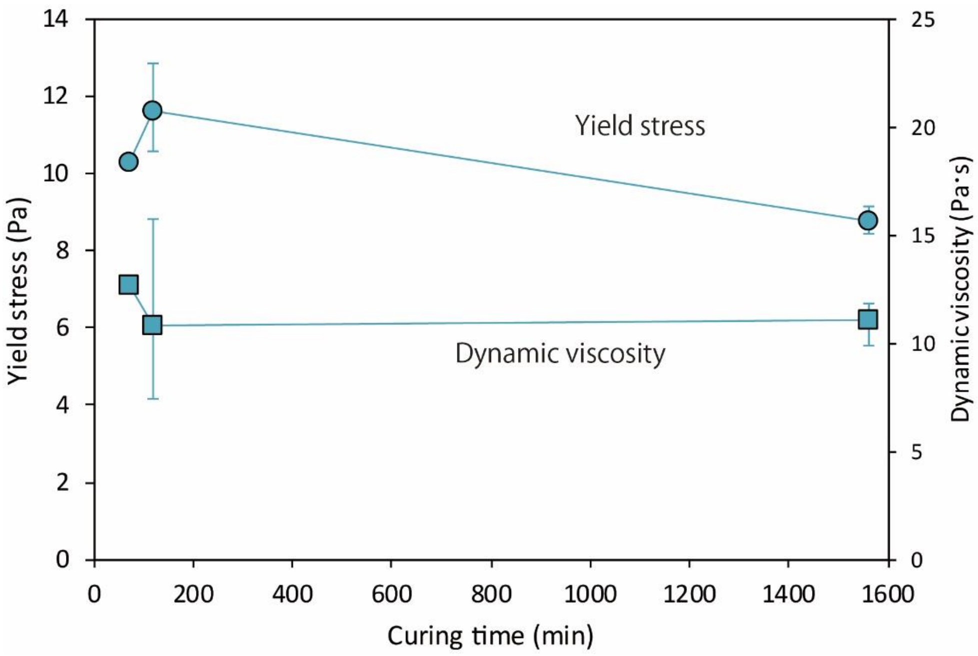

Figure S1 | Effect of curing time on the rheological parameters from the flow ramp test (K40Q60_0.31*Φ* with 0.5% xanthan gum). Error bars denote the maximum and minimum values in three repeated tests.
